# Green Tea Kombucha Impacts Inflammation and Salivary Microbiota in Individuals with Excess Body Weight: A Randomized Controlled Trial

**DOI:** 10.3390/nu16183186

**Published:** 2024-09-20

**Authors:** Gabriela Macedo Fraiz, Dandara Baia Bonifácio, Udielle Vermelho Lacerda, Rodrigo Rezende Cardoso, Viviana Corich, Alessio Giacomini, Hércia Stampini Duarte Martino, Sergio Esteban Echeverría, Frederico Augusto Ribeiro de Barros, Fermín I. Milagro, Josefina Bressan

**Affiliations:** 1 Department of Nutrition and Health, Universidade Federal de Viçosa, Viçosa 36570-900, Brazil; gabriela.fraiz@ufv.br (G.M.F.); dandara.bonifacio@ufv.br (D.B.B.); hercia@ufv.br (H.S.D.M.); 2 Department of Nutrition, Food Science and Physiology, Centre for Nutrition Research, University of Navarra, 31008 Pamplona, Spainfmilagro@unav.es (F.I.M.); 3 Department of Food and Technology, Universidade Federal de Viçosa, Viçosa 36570-900, Brazil; udielle.lacerda@ufv.br (U.V.L.); rodrigocardoso@ufv.br (R.R.C.); fredbarros@ufv.br (F.A.R.d.B.); 4 Department of Agronomy, Food Natural Resources, and Environment (DAFNAE), Università degli Studi di Padova, 35020 Legnaro, Italy; viviana.corich@unipd.it (V.C.); alessio.giacomini@unipd.it (A.G.); 5 Centro de Investigación Biomédica en Red de la Fisiopatología de la Obesidad y Nutrición (CIBERobn), Institute of Health Carlos III, 28029 Madrid, Spain; 6 Navarra Institute for Health Research (IdiSNA), 31008 Pamplona, Spain

**Keywords:** obesity, cytokines, human microbiome, saliva, fermented foods

## Abstract

Background: Green tea kombucha (GTK) is a fermented beverage with promising health benefits, but few studies proved its impact on human health. Thus, we aimed to investigate the impact of GTK on weight loss, inflammation, and salivary microbiota in individuals with excess body weight. Methods: This is a randomized controlled clinical trial that lasted 10 weeks with two groups of individuals with excess body weight: control (CG; n = 29; caloric restriction) and kombucha (KG; n = 30; caloric restriction + 200 mL GTK). Body composition, anthropometry, saliva, and blood collection were performed in the beginning and end of the intervention. Plasma interleukins were determined by flow cytometry. Salivary microbiota was analyzed by 16S rRNA sequencing. Results: Both groups decreased weight, BMI, and body fat (*p* < 0.001) after the intervention, but there were no differences between groups. The KG reduced lipid accumulation product (LAP) (*p* = 0.029). Both groups decreased IL-1β and IL-8, but IL-6 increased in the CG (*p* = 0.023) compared to the kombucha group. Alpha and beta diversity of salivary microbiota increased in the KG. Moreover, the KG presented lower Bacillota/Bacteroidota ratio (*p* = 0.028), and BMI was positively associated with the Bacillota phylum. Conclusions: GTK did not enhance weight loss, but it decreased the LAP. GTK helped in the inflammatory profile and induced positive changes in oral microbiota composition.

## 1. Introduction

Overweight and obesity have reached epidemic proportions around the globe, affecting all ages and socioeconomic groups [1]. In 2020, the proportion of the global population with overweight and obesity (BMI ≥ 25 kg/m^2^) was 38%, and it is projected that this rate will alarmingly increase up to 51% in 2035 [2].

The growing prevalence of excess weight is disturbing since it is strictly related to several metabolic changes resulting in a worse quality of life, shorter life span, and increased healthcare costs [1,3]. The causes of overweight and obesity are multifactorial and involve various aspects in its physiopathology, such as genetic, environmental, nutritional, socioeconomic, and behavioral [4,5]. The abnormal or elevated fat accumulation in the body impacts the proper function of the adipose tissue with increased lipogenesis that results in adipocyte hyperplasia and hypertrophy, inducing hypoxia and infiltration of immune cells in the stromal vascular fraction, generating a systemic chronic low-grade inflammation [3,6]. In response to the increase of free fatty acids and tissue hypoxia, there is an infiltration of polarized M1 macrophages, which elevates inflammatory cytokine secretion, including tumor necrosis factor (TNF) and interleukins (IL-6, IL-8, IL-1β) [7]. Furthermore, there is a commitment to immunomodulatory function with a reduction of M2 macrophages which leads to the lower secretion of anti inflammatory cytokines, such as IL-4 and IL-10 [7,8].

Excess body weight has also been reported to the dysfunction of the salivary microbiota [9]. People with overweight and obesity have a different profile of bacterial saliva community, with lower species richness and abundance of certain bacteria compared to normal weight individuals [9,10,11]. This altered salivary microbiota has been associated with worse health parameters [9,12]. However, little is known about the factors and metabolic pathways that are related to this imbalance in the oral microbiome of individuals with obesity [11].

In this context, fermented foods have been proposed as an adjuvant in the prevention and treatment of non-communicable chronic diseases (NCCDs), such as excess body weight, because of their composition rich in bioactive compounds linked to health benefits, as well as modulation of microbiota [13,14]. Among them, there is kombucha, a drink made with the infusion of *Camellia sinensis*, normally green or black tea, and sugar, fermented by a symbiotic culture of bacteria and yeasts (SCOBY). The fermentation slowly degrades complex substances, which increases the concentration of phenolic compounds, such as catechins, and produces organic acids [15]. Health benefits associated with kombucha have been evidenced in vitro and in animal models, especially involving antioxidant and anti-inflammatory properties, improving low-grade inflammation and outcomes associated with excess body weight [14,16]. In human health, kombucha has been associated with decreasing gastrointestinal symptoms in individuals with inflammatory bowel disease [17]. Moreover, the beverage was demonstrated to improve cardiometabolic markers, mainly, in the glucose metabolism of healthy adults [18], individuals with type 2 diabetes [19], and individuals with obesity [20]. However, the scarcity of studies evaluating the effect of this drink on human health highlights the great need to prove its therapeutic action, mainly, emphasizing its impact on weight loss, inflammation, and microbiota modulation [21,22]. 

To our knowledge, this is the first study evaluating the effect of green tea kombucha (GTK) in anthropometry parameters, inflammatory markers, and modulation of salivary microbiota. The central hypothesis of this study is that GTK may exhibit probiotic and anti-inflammatory properties, contributing to improved weight loss, body composition and inflammatory parameters in individuals with excess body weight. Furthermore, we aimed to evaluate the impact of GTK consumption on the salivary microbiota since the effect of functional food intake on the oral microbiome is not so explored. To test this hypothesis, we evaluated the effect of the beverage by comparing two groups: energy-restricted diet and energy-restricted diet with GTK.

## 2. Materials and Methods

### 2.1. Recruitment of Participants and Experimental Design

This was a parallel randomized controlled clinical trial with a duration of 10 consecutive weeks. Participants with excess body weight were divided into two groups: energy-restricted diet (CG) and energy-restricted diet with green tea kombucha (KG). The primary outcome was the weight loss parameters and secondly, we evaluated inflammatory cytokines and salivary microbiota.

Participants aged 18–45 years old were recruited through dissemination on social media and local newspapers in Viçosa, Brazil. Participants who expressed interest completed an online pre-screening questionnaire. Those who met the inclusion criteria were invited to a face-to-face screening to investigate clinical, dietary, sociodemographic, anthropometric, and body composition information. They were required to have a Body Mass Index (BMI) of at least 27 kg/m², a high waist circumference (≥80 cm for women; ≥94 cm for men), and elevated body fat by Dual-energy X-ray Absorptiometry (DEXA) >30% for women and >25% for men) [23]. The non-inclusion criteria were: chronic diseases such as diabetes, cancer, cardiovascular or gastrointestinal diseases, HIV-positive, anemia, or other inflammatory or metabolic diseases, such as endometriosis or polycystic ovary syndrome; make regular use of nutritional supplements, anti-inflammatory, anxiolytic and anti-depression drugs, corticosteroids, or other drugs that affect lipid or glucose metabolism; those who used antibiotics less than 3 months before the beginning of the study or have had allergic episodes or infectious in the last month, including SARS-CoV-2; participants that were under a weight-loss diet or that did not have a stable weight in the last 3 months (±3% of usual weight); already had the habit of consuming teas, mainly green, black and white teas and/or any fermented foods (such as kefir, fermented milk, kimchi, tempeh, natto, sauerkraut), including kombucha; had aversion to kombucha; usually had alcohol intake greater than >21 alcohol units/week for men and >14 alcohol units/week for women or present episodic binge drinking greater than 10 units for men and greater than 7 units for women at a single time; were smokers, pregnant, or lactating women.

After screening, a one-week run-in period was applied to identify and exclude participants most likely to not adhere to the research protocol. During this run-in week, they had to maintain their usual physical activity and dietary pattern without the intake of fermented foods, teas, olive oil, nuts, and alcoholic beverages. They were not able to take any nutritional supplement, use anti-inflammatory and/or antibiotic drugs, and smoke. At the end of the seven days, those who reported not being able to adhere to the recommendations or who showed a variation greater than 2% of their usual body weight were excluded.

**Figure 1:**
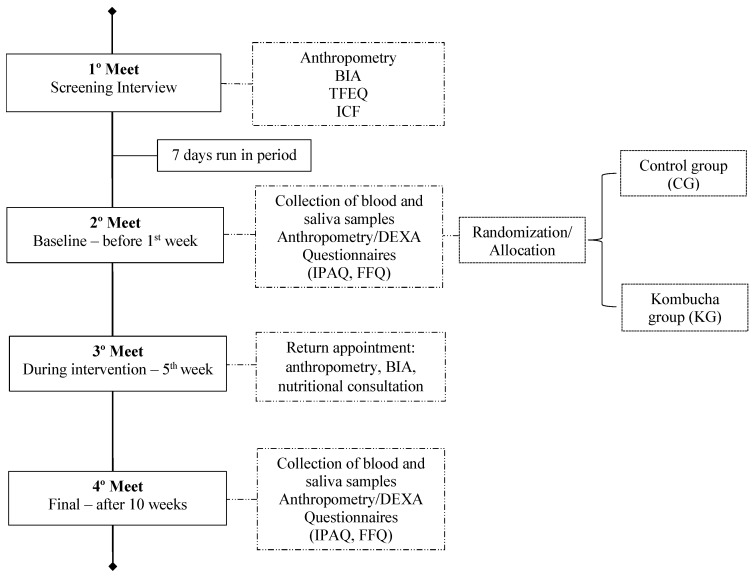
Illustration of the study experimental design. This is a randomized controlled trial that lasted 10 weeks involving individuals with excess body weight allocated in two groups (Control, CG; Kombucha, KG). All participants were screened and those who met the inclusion criteria had to accomplish a run-in period of 7 days. After this, the participants signed the Informed Consent Form (ICF) and initiated the intervention with the data collection (anthropometry, body composition, questionnaires, and biological samples) at baseline and end of study. In the 5th week they had a nutritional return appointment for intervention compliance. BIA: Bioimpedance; DEXA: Dual-energy X-ray Absorptiometry; FFQ: Food Frequency Questionnaire; IPAQ: International Physical Activity Questionnaire; TFEQ: Three Factor Eating Questionnaire.

Then, the selected participants were allocated to one of the two groups using the MinimPy software, version 2.0 (Copyright, Mahmoud Saghaei, 2010–2011) through the stratified minimization method based on BMI, age, and sex, to ensure the balanced distribution of potential factors that interfere with the outcome variables. They were evaluated at the beginning (baseline) and end of the study (10th week) to compare the data from these two moments. The assessments performed were food and physical activity questionnaires, anthropometry, body composition and collection of blood, and saliva samples (Figure 1).

### 2.2. Ethical Aspects

This project was approved (CAAE: 25880819.3.0000.5153; report number: 6.197.412) by the Human Research Ethics Committee of the Federal University of Viçosa (UFV). The procedures described were established under Resolution CNS 466/2012 and the Declaration of Helsinki. This project was also registered and approved (number: RBR-9832wsx) in the Brazilian Registry of Clinical Trials (REBEC). All participants signed the Informed Consent Form (ICF) before the intervention and were enlightened about the benefits and risks inherent to the research, aware that they could withdraw at any time they wanted without any negative repercussions. The researchers kept the participants’ identification confidential, and the data will be archived for five years after the end of the research.

### 2.3. Sample Size

To determine the sample size, G*Power 3.1 software was used [24] with an effect size of 0.7 between two independent means in the Δ values of body fat (kg) [25]. A significance level of 5% and a power of 80% were considered. The minimum sample size generated was 31 participants per group; when adding a 20% loss, the final value for each group was 37 individuals.

### 2.4. Kombucha

#### 2.4.1. Kombucha Production and Intervention

The green tea kombucha was produced in the Cereal Chemistry and Technology Laboratory in the Department of Food Technology-UFV. The beverage was in accordance with the parameters established by the Ministry of Agriculture, Livestock, and Supply (MAPA) [26]. The production of kombucha used 12 g of Brazilian green tea from a certified company (Helio Amaya Cia Ltda, Registro, SP, Brazil) and 50 g of crystal sugar for each liter of mineral water. For the green tea selection, three different brands of the local market were tested and evaluated according to the analysis of the concentration of total phenolics determined by the colorimetric method of Folin–Ciocalteu, using gallic acid as standard [27]. Absorbance was measured in a spectrophotometer at 760 nm and the results were expressed in mg of gallic acid equivalent per mL of tea (mg GAE/mL). The one with the highest total phenolic compounds content was chosen.

**Figure 2:**
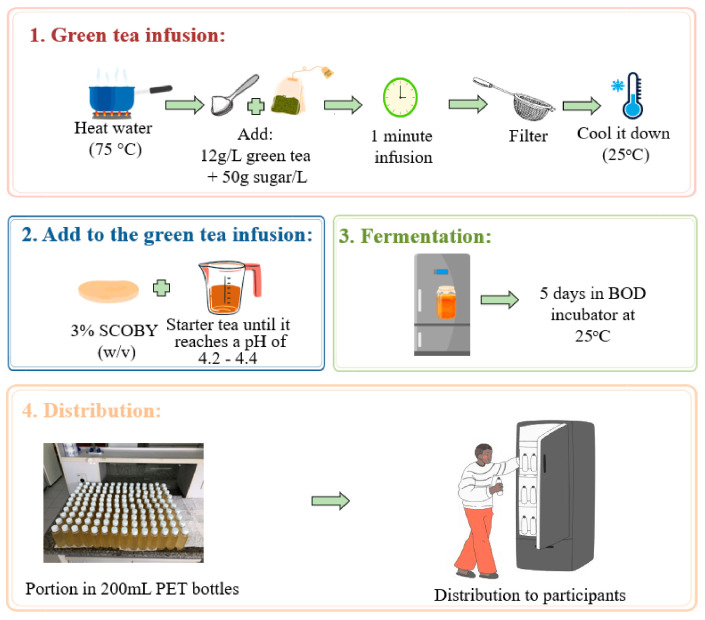
Kombucha production step by step. First, 12 g/L of green tea and 50 g/L of crystal sugar were infused for 1 min after the water reached 70 °C. The tea was filtered and cooled in an ice bath until it reached 25 °C. Subsequently, 3% of Symbiotic Culture of Bacteria and Yeasts (SCOBY) (*w*/*v*) was added to the total volume of green tea produced. A previously produced kombucha was also added to the beverage to reduce the pH until it reaches 4.2 –4.4 to inhibit the growth of pathogenic microorganisms. Ultimately, the buckets with the final preparation were kept inside a Biochemical Oxygen Demand (BOD) incubator for temperature control (25 °C) to ferment for 5 days. After fermentation, the kombucha was portioned in PET bottles in a volume of 200 mL and stored in a refrigerator until distribution to the participants.

In summary, the green tea leaves were infused for 1 min after the water reached 70 °C. Then, the tea was filtered and cooled in an ice bath until it reached 25 °C. The tea was transferred to polypropylene buckets with a capacity of 20 L. Subsequently, 3% of SCOBY (*w*/*v*), obtained from a certified company (Enziquímica Produtos Químicos Ltda, Gravataí, Brazil), was added to the total volume of green tea produced. A previously produced kombucha was also added to the beverage to reduce the pH until it reached 4.2–4.4 to inhibit the growth of pathogenic microorganisms. Ultimately, the buckets with the final preparation were kept inside a Biochemical Oxygen Demand (BOD) incubator for temperature control (25 °C) to ferment for 5 days. After fermentation, the kombucha was portioned in PET bottles in a volume of 200 mL and stored in a refrigerator until distribution to the participants (Figure 2).

The individuals allocated to the KG were provided with seven bottles per week; they had to keep the bottles under refrigeration. Participants were instructed to consume one bottle per day, preferably at lunch or with food. This recommendation was considered to prevent the lower gastric pH during fasting from decreasing the bioavailability of bacteria and yeast contained in kombucha. All participants were oriented not to consume any other fermented food, teas, probiotics, prebiotics, or symbiotics supplements.

#### 2.4.2. Kombucha Composition

The GTK final physicochemical composition is described in Table 1. Description of the methods used to characterize the GTK can be found in previous study [20] and in Appendix A. In each bottle of kombucha (200 mL) consumed daily, there were 3.96 × 10^9^ of lactic acid bacteria, 2.14 × 10^9^ of acetic acid bacteria, and 3.14 × 10^9^ of yeasts. A total of 92 phenolic compounds were present in the green tea kombucha, and the ten most abundant were (−)-epigallocatechin 3-O-gallate, (+)-gallocatechin, catechin 5-O-gallate, epicatechin, (−)-epigallocatechin, 5-O-galloylquinic acid, catechin, 4-*p*-coumaroylquinic acid, quercetin 3-O-rutinoside, and myricetin 3-O-glucoside (Figure S1—Supplementary Materials).

### 2.5. Food Plan and Caloric Restriction

Individuals of both groups received a food plan with a reduction of 500 kcal from their daily caloric requirement estimated using the Estimated Energy Requirement—EER [28]. The food plan consisted of five different menus calculated based on the Brazilian Institute of Geography and Statistics (IBGE) 2008–2009 food tables [29]. In Supplementary Materials Figure S2 there is an example of a 1500 kcal menu. The distribution of macronutrients was based on the recommendations of guidelines for obesity management (55% carbohydrates, 30% lipids, 15% proteins) [30]. In addition, they received qualitative nutritional guidance regarding the healthy dish model, level of food processing, and orientations on how to better choose food with shopping tips. Moreover, they received a document with all the recipes prescribed in the food plans. They were encouraged not to change the foods or meals between the menus.

### 2.6. Assessments and Measurements

#### 2.6.1. Anthropometry and Body Composition

The evaluation of anthropometric parameters was performed at the Laboratory of Energy Metabolism and Body Composition (LAMECC) during screening, in the 1st, 5th, and 10th week of treatment. Participants were weighed using a digital electronic scale (InBody^®^, model 230, Biospace Corp., Seoul, Republic of Korea), wearing light clothes. Height was determined using a vertical stadiometer in millimeters fixed to the wall (SECA^®^, model 206, Hamburg, Germany). BMI was calculated using the following formula: weight [kilograms (kg)] ÷ height^2^ [meters (m)]. Waist (WC) and hip (HC) circumferences were measured using a flexible and inelastic measuring tape, following specific protocols [31,32]. Body composition was analyzed by DEXA (Lunar Prodigy Advance 16 DXA System, version 13.31, General Electric Healthcare, Madison, WI, USA) at Health Division-UFV on the same day of blood collection, after an overnight fast of 10 h at the beginning and end of the intervention. Total fat mass and the fat mass of the android, gynoid, and trunk regions were considered.

Other indexes that are sensitive to abdominal fat and body shape were also performed, including waist–hip ratio (WHR), waist-to-height ratio (WtHR), abdominal volume index (AVI) [33], conicity-index (*C*-index) [34], body roundness index (BRI) [35], body shape index (ABSI) [36], and lipid accumulation product (LAP) [37].

#### 2.6.2. Physical Activity and Food Consumption Questionnaires

Physical activity pattern was assessed at the beginning and end of the study using the International Physical Activity Questionnaire (IPAQ) validated for the Brazilian population [38]. Participants had to maintain the pattern of physical activity throughout the study.

Eating behavior was evaluated in the screening to exclude individuals with possible disturbing eating behavior that could impact during the intervention through the 21-item Three-Factor Eating Behavior Questionnaire (TFEQ-R21), translated and validated for the Brazilian population [39].

Food consumption was assessed by trained nutritionists through food frequency questionnaires (FFQs) that analyzed the individual’s usual food intake before the start of intervention and after it, considering the consumption during the last 10 weeks. All the content of food consumption was transformed into mass (grams) and/or volume (milliliters) through the ERICA-REC24h software (version 24_05_2022) [40]. When any food or food preparation was not included in this database, the Brazilian Table of Food Composition (TBCA) from the University of São Paulo (USP) was used [41].

#### 2.6.3. Inflammation Markers

Blood samples were collected at baseline and 10th week by a qualified nursing technician at the Clinical Analysis Laboratory of the Health Division-UFV (LACDSA). Plasma cytokine concentrations (IL-6, IL-10, IL-8, TNF- α, IL-1β, IL-12p70) were determined by flow cytometry through the BD Accuri™ C6 Plus Flow Cytometer (BD Biosciences, San Jose, CA, USA), using a commercial kit (Cytometric Bead Array CBA Human Th1/Th2/ Th17 Kit, BD Biosciences, USA), according to the manufacturer’s instructions. Data were analyzed using FCAP Array Software v3.0 (BD Biosciences, USA). *C*-reactive protein (CRP) was analyzed with a Mindray/BS-200^®^ Chemistry Analyzer (Shenzhen, China) following the methodology of the commercial kit (Bioclin Bioquímica, Belo Horizonte, Minas Gerais, Brazil).

#### 2.6.4. Saliva Collection and Microbiota Analysis

An unstimulated saliva sample was collected through a 5 mL spontaneous spit into a sterile container. Participants were oriented not to drink or eat at least eight hours before sampling. After collection (within ≤5 min), the saliva was aliquoted and stored at −80 °C (Thermo Fisher Scientific, Waltham, MA, USA/Forma 900 Series^®^) until further analysis.

Genomic DNA was extracted at the Experimental Nutrition Laboratory (Nutrition and Health Department-UFV, Brazil) using QIAamp^®^ DNA Mini Kit (Qiagen, Hilden, Germany), following the manufacturer’s protocol. DNA purity was evaluated via A260/A280 ratio using a NanoDrop 7000 Spectrophotometer (Thermo Fisher Scientific, Waltham, MA, USA), and DNA integrity was checked by 1% agarose gel electrophoresis. Once DNA extraction and purification was performed, it was sent by mail to the University of Illinois, Chicago at the Genome Research Core for amplicon preparation and sequencing. PCR amplification of the V4 region of the prokaryotic 16S rRNA (ribosomal Ribonucleic Acid) gene sequences was performed using Next Generation Sequencing (NGS) with the universal primers (samples: 16S-U515F and 16S-U806R). All PCR products were sequenced on an Illumina NovaSeq6000 instrument according to the standard protocols (Illumina, Inc., San Diego, CA, USA). The PCR program experienced initial denaturation at 95 °C for 5 min; 25 cycles of denaturation at 95 °C for 30 s, annealing at 56 °C for 30 s, extension at 72 °C for 40 s, and final extension of 72 °C for 10 min. To verify that the specific primers had been correctly attached to the samples, 1 μL of the PCR product was checked on a Bioanalyzer DNA 1000 chip (Agilent Technologies, Santa Clara, CA, USA) with an expected size of ≈550 bp. The amplicon mixture was pooled according to the manufacturer’s instructions (Illumina, Inc., San Diego, CA, USA).

16S data processing and bioinformatics were performed considering the Quantitative Insights Into Microbial Ecology (QIIME2 v.2020.8; http://www.qiime2.org, accessed on 1 March 2024) pipeline for raw sequencing data analysis [42]. Paired V4 16S rRNA sequences were trimmed using cutadapt [43] plugin in QIIME2. Sequences were merged, filtered to remove low-quality reads and chimeric sequences, and assigned to amplicon sequence variants (ASVs) using dada2 [44] plugin in QIIME2. Taxonomies were assigned using the Ribosomal Database Project (RDP) classifier (Version 2.14).

Alpha diversity was assessed by the Chao 1, and Simpson indices and Student’s *t*-test were used for alpha diversity comparisons. To visualize the beta diversity between groups, the Bray–Curtis dissimilarity index was calculated and plotted via principal coordinate analysis (PCoA); PERMANOVA was used to test significance. All these analyses were performed using Microbiome Analyst (version 2.0; https://www.microbiomeanalyst.ca/, accessed on 1 March 2024). Differential analysis and Bacillota/Bacteroidota ratio between groups was performed using ALDEx2 (ANOVA-like differential expression analysis for compositional data) package in R, version 4.3.3 [45] and Wilcoxon rank-sum test, respectively. Significance was determined by a 0.1 threshold for FDR corrected *p*-values. Association of taxon abundances with metadata was performed at phylum, genus, and species by using a Generalized Linear Model from the ALDEx2 package, adjusting for group, visit, age, and sex.

### 2.7. Statistical Analyses

To assess the normality of the quantitative variables Shapiro–Wilk test, histograms and boxplots were used. The paired *t*-test was established to compare differences between baseline and final intervention across the same group when the variables presented normal distribution, and the Wilcoxon test was used for variables without normal distribution. For comparisons between groups, independent *t*-tests for normal distribution or Mann–Whitney for non-distribution were used. Results were expressed in mean (standard deviation) for normal distribution or median [percentile (p) 25–75] for variables without normal distribution. The database was created using Microsoft Office Excel version 16.49, 2021©. Analyzes were performed using SPSS software (IBM Corp. Released 2017, NY, USA IBM SPSS Statistics for Windows, Version 25.0. IBM Corp., Armonk, NY, USA). A statistical significance level (α) of 0.05 was adopted for all analyses.

## 3. Results

### 3.1. Participants

**Figure 3:**
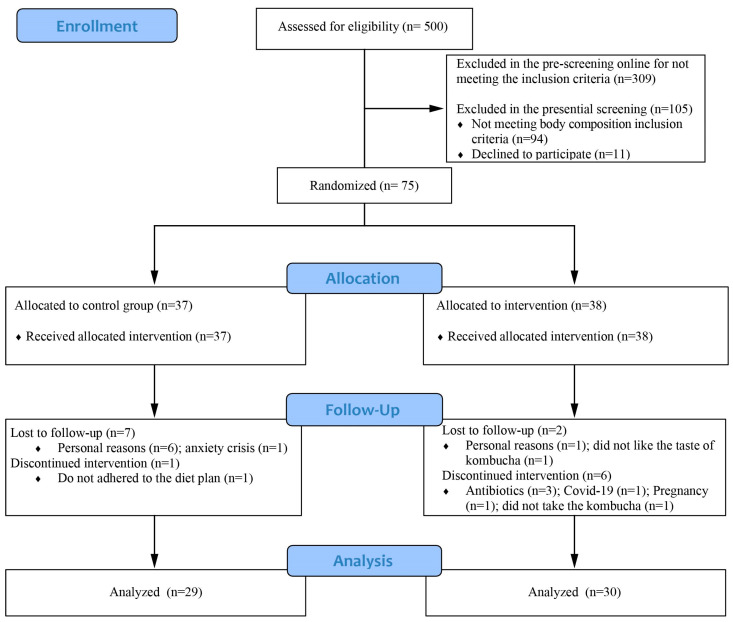
CONSORT flowchart of participants. A total of 500 individuals responded to the online pre-screening form. Of them, 191 accomplished the pre-screening eligibility criteria and were invited to screening. After the presential screening, 105 were excluded. Finally, a total of 75 participants were randomized in the allocation groups (CG = 37 individuals; KG = 38 individuals). In the CG, seven individuals dropped off the study and one was excluded, and in the KG, two lost the follow up and six discontinued the intervention. The data from the excluded participants were not considered in the statistical analysis. In total, 59 individuals completed the study and were analyzed, 29 in the CG and 30 in the KG.

A total of 500 individuals responded to the online pre-screening form. Of them, 191 accomplished the pre-screening eligibility criteria and were invited to attend LAMMEC between March and June of 2023. After the presential screening, 105 were excluded, most of them because of not meeting the body composition criteria (n = 94), and the others declined to participate (n = 11). Finally, a total of 75 participants were randomized in the allocation groups (CG = 37 individuals; KG = 38 individuals). In the CG, seven individuals dropped off the study, and one was excluded due to not adhering properly to the diet; in the KG, two lost the follow up six discontinued the intervention because of antibiotics use (n = 3), confirmed infection of SARS-CoV-2 (n = 1), or pregnancy (n = 1), and one withdrew due to not taking the kombucha for more than three consecutive days. The data from the excluded participants were not considered in the statistical analysis. In total, 59 individuals completed the study and were analyzed, 29 in the CG and 30 in the KG (Figure 3).

### 3.2. Anthropometric and Body Composition

The anthropometric and body composition data of the participants are shown in Table 2. Before the intervention, no significant differences were found between the groups except for WHtR and *C*-index, which were lower in the CG compared to the KG (Table 2). As expected, both groups presented similar age (CG: 32.5 ± 6.9 and KG: 34.7 ± 6.9 years old; *p* = 0.239) and gender distribution (CG: 17 women/12 men, KG: 18 women/12 men; *p* = 0.914).

After the intervention, almost all the anthropometric parameters improved in both groups. The CG lost 2.29 kg (±2.99; *p* < 0.001) and the KG 3.02 kg (±2.09; *p* < 0.001), with no significant differences between groups (*p* = 0.282). DEXA data show that both groups decreased total body fat in all body regions, including trunk, android, and gynoid. Between the anthropometric indices calculated, WHR did not change with the intervention in either group, and AVI, *C*-index, BRI, ABSI, and WHtR decreased equally in the groups. However, only the KG presented a significant reduction in LAP: −8.7 (p25–p75: −15.8–1.1; *p* = 0.029) (Table 2).

### 3.3. Food Intake and Physical Activity

Results related to food intake and physical activity are shown in Table 3. As expected, both groups presented lower energy (kcal) consumption; however a reduction of more than 500 kcal/d was surprisingly reported. We believe the participants underestimated their food intake, behavior that can happen with the FFQ instrument and also in participants under a dietary restriction intervention [46].

No other changes were observed in the distribution of macronutrients (%) intake intra- and inter-groups. The KG increased the fiber (g) intake in relation to the beginning of the study (Δ:2.9 ± 5.4; *p* = 0.018), but this higher consumption was not statistically significant when compared with the CG (*p* = 0.265). The physical activity intensity did not change over the clinical trial (Table 3).

### 3.4. Inflammatory Markers

**Figure 4:**
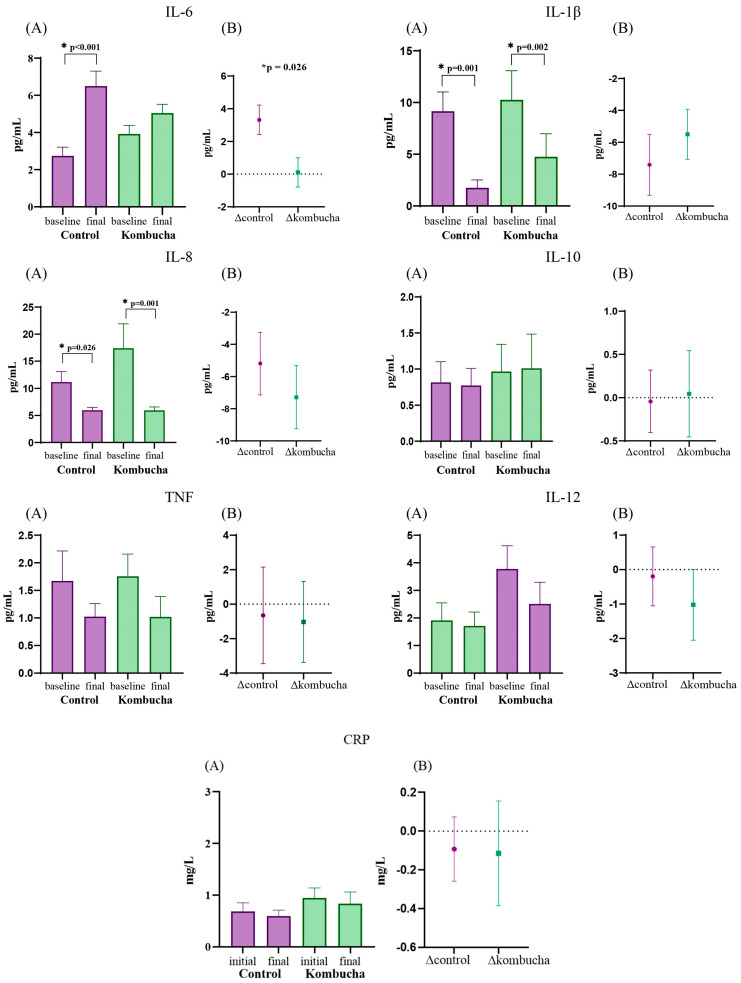
Effect of kombucha intake on inflammatory markers. Values expressed as means (SEM). (**A**): inter and intra-group comparison, only significant *p* value (<0.05). (**B**): comparison of changes between groups after the intervention. CRP: *C*-reactive protein; IL: interleukin. *: values with statistically significant difference After the intervention, no statistically significant changes between control and kombucha groups were observed in IL-1β and IL-8, but these cytokines decreased in both groups when comparing with baseline. The IL-6 increased significantly only in the control group, and this worsening was statistically significant compared with the kombucha group. No differences were found in IL-10, TNF, IL-12 and CRP.

Regarding the inflammatory markers, no differences in baseline were found between groups. Although no statistically significant changes between groups were observed in IL-1β (Δ CG: −7.4 pg/mL vs. Δ KG: −5.5 pg/mL; *p* = 0.586) and IL-8 (Δ CG: −5.2 pg/mL vs. Δ KG: −7.3 pg/mL; *p* = 0.501), these cytokines decreased in both groups when comparing with the baseline. The pro-inflammatory cytokine IL-6 increased significantly only in the CG (*p* < 0.001), and this worsening was statistically significant compared with the KG (Δ CG: 3.3 pg/mL vs. Δ KG: 1.1 pg/mL; *p* = 0.023). No differences were found in the other inflammatory parameters (Figure 4).

### 3.5. Salivary Microbiota

Alpha diversity measured by Chao 1 was significantly higher in the KG after intervention at genus level (*p* = 0.028). At species level, it was also higher but both in the baseline (*p* = 0.031) and after intervention (*p* = 0.001). The Simpson’s diversity index was higher in the CG after the intervention at order (*p* = 0.008), class (*p* = 0.007) and phylum (*p* = 0.034) levels, although these differences were also significant at baseline. Beta diversity by Bray–Curtis dissimilarity was different between groups at species level after the intervention (*p* = 0.017).

**Figure 5:**
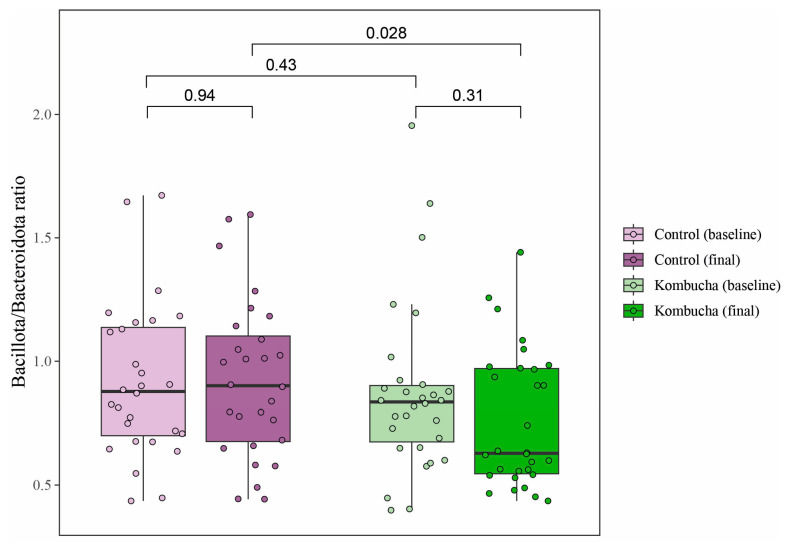
Effect of kombucha intake on the Bacillota/Bacteroidota (namely Firmicutes/Bacteroidetes) ratio within and between groups after intervention. The Bacillota/Bacteroidota ratio was smaller in the kombucha group than in the control group after the treatment. Comparison performed using Wilcoxon rank-sum test from ALDEx2 package in R.

Differential abundance analysis between the two groups conducted at species level showed that *Catonella morbi*, *Schaalia odontolytica*, *Lachnoanaerobaculum umeaense*, *Eubacterium sulci*, *Megasphaera micronuciformis*, *Veillonella dispar*, *Oribacterium sinus*, *Prevotella pallens*, and *Lancefieldella parvula* were less abundant in the KG compared to CG. Some changes at the genus, family, class, and phylum levels were also found (majority FDR < 0.1; Table 4), all of them lower in the KG. The Bacillota-to-Bacteroidota (namely Firmicutes/Bacteroidetes) ratio was smaller in the KG than in the CG after the treatment (*p* = 0.028) (Figure 5).

**Figure 6:**
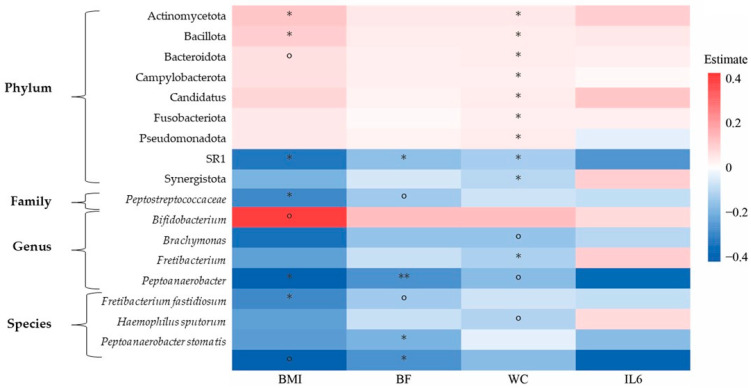
Heat plot showing associations between changes in microbial phyla, genera and species and clinical variables by Generalized Linear Model from ALDEx2 package, adjusting for group, visit, age, sex and a random effect for individual variation; adjusted *p* value denoted by ** < 0.01, * < 0.05, o < 0.1. The inflammatory cytokine IL-6 was not significantly correlated with any of the bacteria. The BMI and WC were positively associated with Bacillota phylum. BF: Body Fat; BMI: Body Mass Index; WC: Waist Circumference.

Associations between bacteria abundance and BMI, WC, IL-6 and BF are shown in Figure 6. The inflammatory cytokine IL-6 was not significantly correlated with any of the bacteria; however, BMI was positively associated with Bacillota phylum (Figure 6).

## 4. Discussion

Kombucha is considered an ancient beverage, but it has gained huge popularity just in the last few years and has been sold as a functional product with promising health benefits [21]. However, little is known about its real impact on human health [21,22]. In this manner, we strive to investigate the effect of the beverage on health parameters in individuals with obesity under an energy-restricted diet. Our hypothesis of primary outcome was partially rejected since the intake of GTK did not enhance weight loss nor body composition and anthropometric changes when compared with the control group. Nevertheless, the LAP, a clinical index used to estimate the risk of metabolic disorders related to cardiovascular disease and type 2 diabetes, significantly decreased in the KG compared to CG. However, the results of this study did confirm our secondary hypothesis since kombucha prevented the significant increase in IL-6 that was observed in the CG, and positively impacted the oral microbiota diversity and abundance.

In fact, several in vitro and animal model studies evidenced antioxidant and probiotic properties of kombucha [14,47], but, to our knowledge, there are only four clinical trials that support the improvement of health in humans. One study observed that black tea kombucha appears to benefit the metabolic health of individuals with obesity by reducing key cardiometabolic markers [20]. Two studies concluded that kombucha ameliorated glucose metabolism acutely [18] and chronically [19]. Moreover, kombucha seems to improve gastrointestinal symptoms in individuals with inflammatory bowel disease [17].

The components of kombucha composition could be associated with the improvement in the parameters studied. The fermentation process enriches the tea’s nutritional composition, resulting in a beverage rich in bioactive compounds [15]. The majority of phenolic compounds present in the kombucha given to the participants of this clinical trial belonged to the class of flavonoids (71%), followed by phenolic acids (25%), lignans (2%), and other polyphenols (1%). It is extremely necessary to consider that the final phenolic profile of the beverage is impacted by the type of tea; the more traditional ones are black and green, but it can also be made with oolong, white, and red tea (Pu-Erh) [48]. Along with the type of tea, other parameters that affect the presence and quantity of bioactive compounds in kombucha are the microorganism’s consortium of the SCOBY, the duration and temperature of fermentation, and the sucrose content [49]. Green tea kombucha has metabolites from the flavonoid class, such as epigallocatechin, epicatechins, epigallocatechin gallate, and epicatechin gallate, and phenolic acids, such as gallic acid [50]. On the other hand, kombucha made with black tea has a higher amount of theaflavins and thearubigins [51,52].

We believe that the phenolic profile of the green tea kombucha is responsible for the changes observed after the intervention. A possible mechanism that could be involved in this scenario is the activation of the sirtuin (SIRT)-1-AMP activated protein kinase (AMPK)-peroxisome proliferator-activated receptor gamma coactivator (PGC)-1α metabolic axis [14]. Bae et al. (2018) observed that epigallocatechin-3-gallate-rich green tea extract supplementation reduced body weight gain and prevented hepatic fat accumulation in mice fed a high-fat diet by upregulation of SIRT-1 and AMPK pathway [53]. Although no changes in body weight was observed, the LAP, an index based on WC and TG, significantly decreased in the kombucha group. LAP has been recently considered a valid predictor of hepatic steatosis and nonalcoholic fatty liver disease [54]. In addition, LAP is one of the principal anthropometric factors for predicting dyslipidemia incidence [55] and has been reported to be more efficient than BMI in adults at risk of cardiovascular disease [37].

The activation of SIRT-AMPK-PGC1α by phenolic compounds can also contribute to the inhibition of the transcription of nuclear factor-κB (NF-κB), and in this manner decreases the synthesis of inflammatory cytokines [56,57]. Indeed, Wang et al. (2021) demonstrated that green tea kombucha was able to inhibit NF-κB signaling, reducing the levels of TNF, IL-1β and IL-6 in sepsis mice challenged with lipopolysaccharide (LPS). The authors emphasized kombucha as an emergent anti-inflammatory drink against systemic inflammation responses [58]. Haghmorad et al. (2020) observed that the improvement in the defense system after kombucha treatment was related to the increased production of Th2 and Treg cytokines (IL-4 and TGF-β) and lower production of Th1 and Th17 cytokines (IFN-γ and IL-17) in experimental autoimmune encephalomyelitis-induced mice [59]. Furthermore, a randomized prospective study with healthy individuals compared the impact of a diet rich in plant-based fiber with one high in fermented foods, including kombucha; they observed an improvement in the immune response with a decrease in the concentration of 19 cytokines, chemokines, and other serum proteins, including IL-6, along with increased gut microbiota diversity in the group that consumed fermented foods [60]. The authors suggested that fermented foods may be powerful modulators of the human microbiome and immune system axis and essential to combat non-communicable diseases, such as obesity [60].

Surprisingly, in this study the IL-6 increased in both groups, but only significantly in the control group. This may indicate that the antioxidant and anti-inflammatory effect of kombucha possibly counteracted the rise in this cytokine in individuals who were under an energy-restricted diet. We also hypothesized that probably the caloric restriction and weight loss was responsible for the increase of IL-6. Previous studies have shown that energy-allocation and lipid mobilization may increase this cytokine [61,62], although it has been also commonly described that weight loss through energy restricted diet and/or exercise may decrease IL-6 [63,64].

In addition to phenolic compounds, other substances may be related to the impact of green tea kombucha on participants’ health. Among them, there are organic acids, such as gluconic acid (GA), glucuronic (GlcUA), acetic, and D-saccharic-1,4-lactone (DSL) that confer to kombucha’s potential antibacterial and antioxidant activities that even collaborate in the prevention of beverage contamination by pathogenic bacteria [48,65]. GlcUA is considered a potent natural detoxifier, capable of enhancing glucuronidation of endogenous and xenobiotic metabolites, with subsequent elimination via urine or bile [49,66]. DSL, produced by the bacteria Gluconacetobacter sp. A4 during kombucha fermentation, also presents a high antioxidant capacity, acting mainly by inhibiting beta-glucuronidase, an enzyme associated with generation of toxic and carcinogenic substances via hydrolysis of glucuronides in the intestinal lumen [67]. In animal studies, DSL was shown to be effective in inhibiting oxidative stress and promoting hepatic and kidney protective effects [68].

Both gut and oral microbiomes are important in the performance of individuals’ health, mainly regulating the immune system and metabolic function [9]. Although the mechanisms by which the oral microbiome is related to obesity are far from being explained, there are articles indicating that, like the gut microbiome, the salivary microbiota profile is different in people with excess body weight [9,10,11].

Although the impact of diet and certain foods on gut microbiota are well documented, the effect of food/beverage intake on the oral microbiome is not so explored [69]. Some studies investigated the association between tea intake and oral microbiota [70,71,72]. In accordance with the increased diversity found in our study, Peters et al. (2018) demonstrated that tea consumption was correlated with increased richness, diversity, and changes in the oral microbial community profile [70]. Liu et al. (2020), in contrast, observed a reduction in the bacterial community diversity after daily consumption of 1.0 L of oolong tea (*Camellia sinensis*), but only three healthy individuals were evaluated, which can compromise the statistical analysis [72].

In our results, it was evident that somehow the kombucha changed the salivary microbiota profile with a predominant decrease in the abundance of bacteria at all levels, except for order. Among them, the genus Eubacterium has been associated with chronic periodontitis [73], and *Veillonella* and *Leptotrichia* were found to be more abundant in individuals with metabolic imbalance (diabetes) [74]. In addition, many Prevotella species, including *Prevotella pallens*, have been related to the occurrence of dental caries disease [75] and with the viral load in saliva of patients with COVID-19 [76]. Thus, to such a degree, it can be considered positive that these genera decreased after the kombucha consumption. The antimicrobial activity of kombucha bioactive compounds, such as epigallocatechin gallate and epicatechin gallate, can explain some of the findings [77]. In fact, the use of (-)-epigallocatechin-3-gallate and green tea as mouthwash is an alternative to anti-cariogenic disease [78] and also to chronic periodontitis treatment because of its anti-microbial activity [79].

Obesity and metabolic imbalance are associated with oral inflammation, such as periodontitis, and a higher production of inflammatory cytokines [80]. In this context, we evaluated if some bacteria were related to IL-6 since it was observed that kombucha could prevent the increase of this interleukin, but no associations were found. Otherwise, BMI and Bacillota phylum were positively associated. Qadir and Assafi (2021) observed that individuals with obesity and overweight presented a higher frequency of Bacillota in saliva compared to normal-weight participants. Along with this, the Bacillota/Bacteriodota ratio was higher with an increasing BMI [81]. In fact, the Bacillota/Bacteriodota ratio has been suggested as an important contributing factor in the development of obesity, even though this is a controversial conclusion since phylum is a broader taxonomic level [9,82].

As a major strength, this work adheres to a carefully randomized controlled trial design lasting for 10 weeks. To our knowledge this is the first study investigating the impact of green tea kombucha intake on inflammation markers, body composition, and salivary microbiota in individuals with excess body weight under an energy restricted diet, known as one of the most effective interventions to weight loss. We tried to control the main factors that could be considered as confounders, such as physical activity, alcohol consumption, smoking, drug intake, and dietary patterns. Nevertheless, our study has some limitations such as a limited sample size and the self-report of physical activity and food consumption, which can be underestimated in participants with excess weight under energy restricted diet [83]. Furthermore, the oral health of participants was not assessed, which may be a confounder in the salivary microbiota. In addition to that, the blinding of intervention was not possible, and a higher dosage of kombucha may have been necessary to enhance the effects on weight loss.

## 5. Conclusions

In conclusion, although green tea kombucha consumption in the context of a healthy energy-restricted diet did not significantly change the conventional anthropometric parameters, it directly impacted the lipid accumulation product in the kombucha group, reducing it by 4.6-fold compared to the control group. Furthermore, the control group presented a higher increase in the interleukin-6 compared to the kombucha group (Δ control group: 3.3 pg/mL vs. Δ kombucha group: 1.1 pg/mL). This emphasizes that the bioactive effect of green tea kombucha may be avoided by the significant worsening of this inflammatory cytokine.

In addition, the beverage induced positive alterations in the salivary microbiota composition, decreasing pathogenic species. Moreover, green tea kombucha impacted richness, mainly by Chao 1 index at genus level, and beta-diversity through Bray–Curtis dissimilarity at species level.

Summary, the intake of green tea kombucha did not detrimentally affect the health of individuals with excess body weight, suggesting it as an option drink to be prescribed in weight loss strategies.

## Figures and Tables

**Table 1:** Green tea kombucha characterization.

Components	Concentration
Sucrose, g/L	22.24
Fructose, g/L	12.57
Glucose, g/L	11.49
Acetic acid, g/L	3.43
Ethanol, g/L	4.7
pH	3.41 ± 0.09
Total acidity, *w*/*v* acetic acid	0.20 ± 0.02
Lactic acid bacteria, CFU/mL	1.98 × 10^7^
Acetic acid bacteria, CFU/mL	1.07 × 10^7^
Yeast, CFU/mL	1.57 × 10^7^
Total phenolics (Folin–Ciocalteu), mg GAE/mL	0.32
Antioxidant capacity (ABTS assay), μmol TE/mL	3.24

GAE: Gallic acid equivalent; TE: Trolox equivalent.

**Table 2:** Comparison of anthropometry and body composition parameters before and after intervention of participants with excess body weight, according to allocation groups.

Variables	CG (n = 29)				KG (n = 30)					
	Baseline	After 10 Weeks	Δ	* p-Value	Baseline	After 10 Weeks	Δ	* p-Value	# *p*-Value	Δ *p*-Value
Anthropometry										
Weight, kg	91.47 (13.52)	89.17 (12.48)	−2.29 (2.99)	**<0.001**	92.54 (13.42)	89.52 (13.03)	−3.02 (2.09)	**<0.001**	0.760	0.282
BMI, kg/m^2^	32.34 (3.58)	31.59 (3.79)	−0.76 (1.03)	**<0.001**	33.2 (3.7)	32.0 (3.6)	−1.2 (0.75)	**<0.001**	0.403	0.167
WC, cm	92.8 (8.6)	90.2 (7.2)	−2.5 (3.2)	**<0.001**	97.2 (−10.4)	94.3 (9.9)	−2.9 (2.7)	**<0.001**	0.078	0.584
HC, cm	114.7 (8.3)	112.8 (7.0)	−1.9 (4.36)	**0.024**	115.4 (7.9)	112.8 (7.88)	−2.6 (2.6)	**<0.001**	0.734	0.431
NC, cm	37.7 (3.7)	36.7 (3.2)	−1.0 (1.3)	**<0.001**	37.7 (3.8)	37.0 (3.9)	−0.7 (0.8)	**<0.001**	0.991	
Waist-to-Height ratio	0.55 (0.04)	0.53 (0.03)	−0.02 (0.01)	**<0.001**	0.58 (0.05)	0.56 (0.05)	−0.02 (0.01)	**<0.001**	**0.015**	0.317
Waist-to-Hip ratio	0.81 (0.07)	0.80 (0.07)	−0.01 (0.02)	0.132	0.84 (0.07)	0.83 (0.07)	−0.01 (0.03)	0.263	0.120	0.811
Conicity-index	1.15 (0.07)	1.13 (0.06)	−0.02 (0.02)	**0.003**	1.19 (0.07)	1.18 (0.07)	−0.01 (0.03)	**0.006**	**0.025**	0.975
ABSI	0.074 (0.005)	0.007 (0.001)	−0.067 (0.010)	**<0.001**.	0.073 (0.004)	0.006 (0.001)	−0.067 (0.004)	**<0.001**	0.648	0.654
AVI	17.77 (3.02)	16.81 (2.44)	−0.95 (1.24)	**<0.001**	19.43 (4.02)	18.28 (3.63)	−1.53 (1.11)	**<0.001**	0.079	0.522
BRI	4.41 (0.74)	4.22 (0.74)	−0.25 (0.30)	**<0.001**	4.71 (0.74)	4.62 (1.01)	−0.34 (0.31)	**<0.001**	0.139	0.251
LAP	34.6 (23.7–68.5)	33.2 (18.0–50.2)	−1.8 (−5.5–2.5)	0.122	46.2 (32.5–73.5)	38.0 (27.2–62.4)	−8.7 (−15.8–1.1)	**<0.001**	0.197	**0.029**
Body composition										
Trunk BF, kg	20.3 (5.1)	18.8 (4.9)	−1.5 (2.1)	**0.001**	22.7 (4.5)	21.0 (4.3)	−1.7 (1.5)	**<0.001**	0.069	0.677
Android BF, kg	3.1 (0.9)	2.8 (0.7)	−0.3 (0.4)	**0.001**	3.5 (0.9)	3.3 (0.9)	−0.2 (0.3)	**0.001**	0.111	0.370
Gynoid BF, kg	7.0 (6.1–8.5)	6.5 (5.5–8.1)	−0.5 (−0.8–−0.1)	**<0.001**	7.3 (6.1–8.3)	6.9 (5.8–8.0)	−0.4 (−0.6–−0.08)	**<0.001**	0.976	0.301
Total BF, kg	37.0 (33.6–44.8)	35.0 (31.0–40.7)	−2.0 (−3.4–0.9)	**<0.001**	39.2 (36.5–45.4)	38.6 (34.0–43.3)	−2.3 (−4.0–−0.7	**<0.001**	0.154	0.756
Trunk MM, kg	23.14 (4.7)	23.15 (4.6)	0.01 (1.50)	0.959	23.45 (4.99)	23.12 (4.88)	−0.3 (1.1)	0.151	0.755	0.360
Android MM, kg	3.1 (2.6–3.9)	3.3 (2.6–3.7)	0.2 (0.3–0.2)	**<0.001**	3.2 (2.7–3.8)	3.2 (2.6–4.0)	0.02 (−0.1–0.2)	**<0.001**	0.994	0.432
Gynoid MM, kg	7.1 (5.9–8.8)	7.6 (5.6–8.5)	−0.1 (−0.5–0.2)	**<0.001**	6.8 (6.1–7.7)	6.6 (5.9–8.8)	0.06 (−0.4–0.4)	**<0.001**	0.773	0.276
Total MM, kg	4.6 (3.9–5.7)	4.9 (3.9–5.7)	−0.1 (−1.7–1.3)	**<0.001**	4.7 (3.9–5.6)	4.4 (3.8–5.7)	−0.3 (−1.3–0.2)	**<0.001**	0.716	0.608
Trunk BF, %	45.5 (6.6)	43.6 (7.9)	−1.8 (3.5)	**0.009**	48.1 (4.6)	46.5 (4.9)	−1.6 (2.2)	**0.001**	0.112	0.677
Android BF, %	47.8 (6.1)	45.2 (7.7)	−2.6 (4.2)	**0.003**	50.8 (5.5)	49.0 (5.8)	−1.8 (2.6)	**0.001**	0.081	0.370
Gynoid BF, %	49.0 (9.3)	47.5 (9.9)	−1.5 (2.3)	**0.002**	49.6 (7.0)	48.4 (7.5)	−1.2 (2.0)	**0.003**	0.879	0.587
Total BF, %	45.0 (5.9)	44.0 (6.1)	−1.0 (2.4)	**<0.001**	44.7 (5.9)	43.5 (6.1)	−1.2 (1.5)	**<0.001**	0.344	0.762

Quantitative data expressed as mean (SD) or median (percentile 25–75). * *p*-value: comparison between baseline and final results across the same group (Paired *t*-test or Wilcoxon); # *p*-value: comparison of baseline values between groups (independent *t*-test or Mann–Whitney test); Δ *p*-value: comparison of Δ (=final − baseline) between groups. Values with statistically significant difference indicated in bold. ABSI: A Body Shape Index; AVI: Abdominal Volume Index; BRI: Body Roundness Index; BMI: Body Mass Index; BF: Body Fat; CG: Control group; HC: Hip Circumference; KG: Kombucha group; LAP: Lipid Accumulation Product; MM: muscle mass; NC: Neck Circumference; WC: Waist Circumference.

**Table 3:** Comparison of food intake and physical activity variables before and after intervention of participants with excess body weight, according to allocation groups.

Variables	CG (n = 29)				KG (n = 30)					
	Baseline	After 10 Weeks	Δ	* p-Value	Baseline	After 10 Weeks	Δ	* p-Value	*# p*-Value	Δ *p*-Value
Food intake										
Energy, kcal	2171.8 (702.5)	1536.9 (436.6)	−634.9 (701.6)	**<0.001**	1914.8 (568.7)	1261.1 (374.1)	−653.5 (514.9)	**<0.001**	0.133	0.797
Carbohydrate, %	47.3 (6.4)	51.5 (24.1)	4.3 (25.5)	0.524	46.3 (7.2)	56.9 (26.6)	10.6 (26.6)	0.178	0.756	0.422
Lipids, %	35.8 (5.6)	34.6 (12.4)	−1.2 (13.1)	0.787	35.4 (6.2)	35.9 (19.3)	−0.5 (21.8)	0.453	0.856	0.705
Protein, %	16.1 (2.5)	18.5 (7.2)	2.4 (7.8)	0.191	16.8 (2.0)	20.2 (10.0)	3.3 (10.4)	0.276	0.152	0.832
Fiber, g	10.3 (1.9)	11.9 (4.1)	1.5 (4.7)	0.145	10.9 (2.7)	13.8 (5.8)	2.9 (5.4)	**0.018**	0.554	0.265
Physical activity										
Walking (METs/min/week)	242.2 (266.5)	212.1 (265.9)	−30.1 (93.6)	0.108	148.5 (222.0)	122.1 (256.9)	−26.4 (122.3)	0.293	0.130	0.899
Moderate (METs/min/week)	470.3 (416.2)	499.3 (452.6)	28.9 (148.1)	0.288	490.5 (587.1)	479.2 (554.9)	−11.3 (239.2)	0.725	0.498	0.442
Vigorous (METs/min/week)	804.1 (631.3)	834.5 (806.2)	30.3 (428.6)	0.783	1072.0 (839.5)	1058.7 (823.4)	−13.3 (251.9)	0.866	0.195	0.634
Total (METs/min/week)	1559.5 (911.5)	1584.1 (1025.3)	24.6 (446.9)	0.769	1711.0 (1044.0)	1659.9 (1076.2)	−51.1 (354.3)	0.435	0.556	0.475

Quantitative data expressed as mean (SD). * *p*-value: comparison between baseline and final results across the same group (Paired *t*-test or Wilcoxon); # *p*-value: comparison of baseline values between groups (independent *t*-test or Mann–Whitney test); Δ *p*-value: comparison of Δ (= final − baseline) between groups. Values with statistically significant difference indicated in bold. CG: Control group; KG: Kombucha group; MET: Metabolic equivalent of task.

**Table 4:** Bacterial taxa statistically significant between individuals with excess body weight in control (n = 29) and kombucha (n = 30) groups after the intervention.

		Effect	*p* Values	FDR	↓/↑
Species	*Catonella morbi*	−1.11978	4.3 × 10^−5^	0.006744	↓kombucha
	*Schaalia odontolytica*	−1.37204	0.000104	0.008858	↓kombucha
	*Lachnoanaerobaculum umeaense*	−0.76314	0.000253	0.014964	↓kombucha
	*Eubacterium sulci*	−0.46172	0.00097	0.042991	↓kombucha
	*Megasphaera micronuciformis*	−0.65994	0.003349	0.09071	↓kombucha
	*Veillonella dispar*	−0.52692	0.003554	0.092012	↓kombucha
	*Oribacterium sinus*	−0.9763	0.003879	0.092454	↓kombucha
	*Prevotella pallens*	−0.44781	0.005143	0.097005	↓kombucha
	*Lancefieldella parvula*	−0.77349	0.006298	0.099679	↓kombucha
	*Lachnospiraceae catonella*	−1.08107	7.97 × 10^−5^	0.005222	↓kombucha
Genus	*Lachnoanaerobaculum*	−0.82378	0.000132	0.006157	↓kombucha
	*Schaalia*	−0.97974	0.000592	0.017186	↓kombucha
	*Eubacterium*	−0.50944	0.001291	0.028349	↓kombucha
	*Leptotrichia*	−0.65952	0.002691	0.046652	↓kombucha
	*Oribacterium*	−0.79468	0.004703	0.066296	↓kombucha
	*Megasphaera*	−0.55551	0.006986	0.081696	↓kombucha
	*Veillonella*	−0.91501	0.007617	0.084506	↓kombucha
	*Segatella*	−0.57846	0.009331	0.093197	↓kombucha
Family	Actinomycetaceae	−1.17493	0.002409	0.066254	↓kombucha
	Eubacteriaceae	−0.61873	0.003868	0.07029	↓kombucha
	Lachnospiraceae	−1.17577	0.004725	0.076341	↓kombucha
Class	Clostridia	−0.74368	0.001192	0.019575	↓kombucha
	Actinobacteria	−0.68961	0.010358	0.082534	↓kombucha
Phylum	Actinomycetota	−0.84915	0.009424	0.059404	↓kombucha
	Bacillota	−0.77054	0.013535	0.060373	↓kombucha
	Fusobacteriota	−0.53552	0.016236	0.063708	↓kombucha
	SR1	0.401023	0.007691	0.084599	↑kombucha

Differential analysis was performed using Wilcoxon rank-sum test from ALDEx2 package in R. FDR cutoff point <0.1. **↑:** increased abundance, **↓:** decreased abundance.

## Data Availability

The data that support the findings of this study are available upon request from the corresponding author. The data are not publicly available since it contains personal content that could compromise the privacy of the research participants.

## Supplementary Materials

The following supporting information can be downloaded at: www.mdpi.com/xxx/s1,; Figure S1: Phenolic compounds found in the green tea kombucha through UPLC-MSE; Figure S2. Example of a 1500 kcal food plan; Table S1: CONSORT Checklist [84].

## Appendix A

A description of the methods (total and volatile acidity, pH, total phenolics, microbiological characterization, and quantification of acetic acid, sugar and ethanol) to characterize the kombucha final physicochemical composition has been previously described elsewhere by our research group [20]. In Appendix A there is a description of the phenolic profile methodology used.

### Phenolic Profile by UPLC-MSE

The phenolic profile was obtained using ultra-performance liquid chromatography coupled with mass spectrometry (UPLC-MSE) and an electrospray ionization source, following the methodology of previous study with minor adjustments [50]. Green tea kombucha was freeze-dried and reconstituted in 3 mL of 2% methanol (LC-MS grade), 5% acetonitrile (LC-MS grade), and 93% Milli-Q water. After filtration through a 0.22 μm PTFE filter (Analytical), the extract was stored in vials. Standards for phenolic compounds (Sigma Aldrich, St. Louis, MI, USA) were prepared at a concentration of 10 ppm and injected three times before sample analysis to ensure consistency. A five μL aliquot of each sample was injected into the UPLC Acquity system (Waters Co., Milford, MA, USA) connected to the Xevo G2S quadrupole time-of-flight (Q-Tof) (Waters Co., Wilmslow, Cheshire, UK) with electrospray ionization and a QTof mass analyzer. Chromatographic separation was conducted using a UPLC HSS T3 C18 column (100 mm × 2.1 mm, 1.8 μm particle diameter; Waters Co., Wilmslow, Cheshire, UK) at 30 °C with a 0.5 mL/min flow rate, using ultrapure water with 0.3% formic acid and 5 mM ammonium formate (mobile phase A); and LC-MS grade acetonitrile containing 0.3% formic acid (mobile phase B). The gradient used was: 0 min −97% A; 11.80 min −50% A; 12.38 min −15% A; 14.23 min −15% A; 14.70 min −97% A. Data were acquired in MS E mode with argon as collision gas, applying low and high collision energy (25 to 55 V). Acquisitions were performed in negative and centroid mode for ions with *m*/*z* 50–1000. Ionization conditions included a cone voltage 30 V, capillary voltage 3.0 kV; desolvation gas (N2) 1200 L/h at 600 °C; cone gas 50 L/h and source temperature at 150 °C. Phenolic compounds were classified before semi-quantification using representative standards. Processed data were exported to XLSTAT software, version 2016.06.35661 (Addinsoft, Paris, France), and abundance values were used for relative quantification. Thestatistical analysis considered was One-way ANOVA, Tukey post-test, *p* < 0.05.
