# Supplementary material for: Green Tea Kombucha Impacts Inflammation and Salivary Microbiota in Individuals with Excess Body Weight: A Randomized Controlled Trial
Source: Nutrients. 2024 Sep 20;16(18):3186. doi: 10.3390/nu16183186 (PMC11435194; doi:10.3390/nu16183186)
Supplement: Supplementary file 1 [file nutrients-16-03186-s001.zip › nutrients-3218348-supplementary.pdf]

Supplementary Material

Figure S1. Phenolic compounds found in the green tea kombucha through UPLC-MS^E.

Name of compound	Molecular formula	*m/z*	RT (min)	Score (%)	FS (%)	Error (ppm)	IS (%)	Class	Abundance
Sesamolinol	C20H20O7	371.1172	0.55	34.4	0.0	9.53	82.51	L	2.01E+04
Dicaffeoylquinic acid isomer 1	C25H24O12	515.1221	0.73	36.2	0.4	5.00	86.29	PA	3.01E+04
6''-O-acetyldaidzin	C23H22O10	457.1181	1.05	34.6	0.0	8.93	82.73	F	2.80E+04
Dicaffeoylquinic acid isomer 2	C25H24O12	515.1237	1.05	34.8	0.2	8.12	82.84	PA	3.60E+04
Sesaminol/sesamolin	C20H18O7	369.1007	1.32	43.4	37.6	7.41	87.83	L	8.99E+03
Scopoletin	C10H8O4	191.0333	1.82	36.3	0.0	-8.71	91.09	OP	9.57E+03
5-O-galloylquinic acid	C14H16O10	343.0664	2.19	49.8	52.4	-1.95	98.84	PA	3.77E+06
Caffeoylquinic acid isomer 1	C16H18O9	353.0868	4.15	37.6	1.6	-2.97	90.08	PA	2.94E+04
Dihydrocaffeic acid	C9H10O4	181.0488	4.51	50.5	65.0	-10.00	98.27	PA	1.84E+05
3-O-methylgallic acid	C8H8O5	183.0282	4.51	41.2	16.3	-9.13	99.66	PA	1.40E+06
Caffeoylquinic acid isomer 2	C16H18O9	353.0863	4.53	44.6	29.2	-4.36	98.84	PA	8.36E+05
**2,5-dihydroxybenzoic acid**	**C7H6O4**	**153.0179**	**5.14**	**46.4**	**42.3**	**-9.00**	**99.66**	**PA**	**6.44E+04**
**4-hydroxybenzoic acid**	**C7H6O3**	**137.0231**	**5.15**	**37.7**	**0.0**	**-9.89**	**99.29**	**PA**	**8.98E+04**
Esculetin	C9H6O4	177.0176	5.18	37.6	1.5	-9.92	97.60	OP	1.91E+04
Esculin	C15H16O9	339.0708	5.18	45.1	35.8	-3.86	94.44	OP	7.43E+04
5-(3',5'-dihydroxyphenyl)-γ-valerolactone	C11H12O4	207.0646	5.19	35.7	0.0	-8.19	87.87	PA	6.06E+03
4-O-methylgallic acid	C8H8O5	183.0282	5.28	37.6	0.0	-9.23	98.39	PA	5.92E+05
Procyanidin dimer B-type isomer 1	C30H26O12	577.1332	5.53	36.0	0.0	-3.44	84.04	F	1.95E+04
**(-)-epigallocatechin**	**C15H14O7**	**305.0653**	**5.66**	**48.3**	**47.1**	**-4.37**	**99.37**	**F**	**1.21E+06**
3,4-dihydroxyphenyllactic acid	C9H10O5	197.0443	5.78	36.5	0.0	-6.45	89.58	PA	6.08E+03
Procyanidin dimer B-type isomer 2	C30H26O12	577.1338	5.78	44.3	33.9	-2.40	90.65	F	2.52E+04
p-coumaric acid 4-O-glucoside	C15H18O8	325.0913	5.82	38.7	0.0	-4.75	99.11	PA	1.12E+05
Apigenin 7-O-glucuronide	C21H18O11	445.0751	5.94	41.6	25.6	-5.70	88.86	F	2.15E+04
Gallic acid ethyl ester	C9H10O5	197.0437	5.98	43.2	26.7	-9.41	99.40	PA	1.44E+06
**(+)-catechin**	**C15H14O6**	**289.0700**	**5.98**	**42.5**	**19.6**	**-5.96**	**99.42**	**F**	**1.73E+06**
Salvianolic acid D	C11H10O6	237.0418	6.00	40.1	11.1	5.44	95.75	OP	6.24E+04
Quercetin 3-O-glucuronide	C21H18O13	477.0656	6.00	38.9	7.1	-3.95	91.91	F	6.74E+03
Gallic acid 3-O-gallate	C14H10O9	321.0235	6.07	38.0	0.0	-5.23	96.09	PA	3.97E+04
Feruloylquinic acid isomer 1	C17H20O9	367.1015	6.08	39.5	6.3	-5.32	97.10	PA	1.73E+05
**Vanillic acid**	**C8H8O4**	**167.0336**	**6.12**	**47.9**	**51.2**	**-8.46**	**97.55**	**PA**	**5.52E+04**
Dimethylmatairesinol	C22H26O6	385.1620	6.16	35.1	0.0	-9.47	85.82	L	1.39E+04
Oleoside dimethylester	C18H26O11	417.1375	6.30	37.4	0.0	-6.66	94.31	OP	7.82E+03
**Caffeic acid**	**C9H8O4**	**179.0337**	**6.37**	**50.8**	**63.5**	**-7.17**	**98.44**	**PA**	**5.20E+05**
Feruloyl glucose	C16H20O9	355.1019	6.46	38.2	0.0	-4.44	96.04	PA	3.90E+04
Eriodictyol 7-O-rutinoside	C27H32O15	595.1664	6.46	52.6	66.4	-0.71	97.45	F	1.61E+05
**Syringic acid**	**C9H10O5**	**197.0442**	**6.60**	**38.2**	**0.0**	**-6.73**	**98.86**	**PA**	**2.59E+05**
Procyanidin dimer B-type isomer 3	C30H26O12	577.1339	6.60	37.7	0.1	-2.13	90.75	F	2.72E+05
Isopimpinellin	C13H10O5	245.0469	6.71	38.3	0.0	5.31	97.76	OP	4.80E+04
Chicoric acid	C22H18O12	473.0718	6.79	38.7	0.0	-1.56	95.32	PA	4.02E+05
Neoeriocitrin	C27H32O15	595.1659	6.82	41.9	17.1	-1.53	94.04	F	3.69E+04
(+)-gallocatechin	C15H14O7	305.0655	6.89	52.6	68.0	-3.86	99.57	F	6.05E+05
4-p-coumaroylquinic acid	C16H18O8	337.0921	6.92	48.8	48.0	-2.35	98.92	PA	2.63E+06
(-)-epicatechin	C15H14O6	289.0708	6.95	50.1	55.3	-3.38	98.99	F	1.39E+06
Feruloylquinic acid isomer 2	C17H20O9	367.1012	6.98	37.1	10.1	-6.11	82.23	PA	1.90E+04
Eriodictyol 7-O-glucoside	C21H22O11	449.1083	6.98	46.5	37.8	-1.45	96.43	F	1.32E+05
Apigenin di-C-glucoside isomer 1	C27H30O15	593.1511	6.98	51.5	59.3	-0.19	98.65	F	4.03E+05
4-hydroxycoumarin	C9H6O3	161.0229	7.08	40.4	12.9	-9.28	99.23	OP	4.27E+04
**Gallic acid**	**C7H6O5**	**169.0128**	**7.08**	**46.4**	**41.9**	**-8.34**	**99.21**	**PA**	**7.48E+05**
5,6,7-trihydroxyflavone	C15H10O5	269.0442	7.08	43.9	32.5	-5.12	92.74	F	1.73E+04
(-)-epigallocatechin 3-O-gallate	C22H18O11	457.0773	7.08	48.8	46.9	-0.82	98.34	F	4.01E+06
3-methoxy-4-hydroxyphenyllactic acid	C10H12O5	211.0598	7.14	46.8	45.2	-6.58	96.22	PA	3.01E+04
Feruloylquinic acid isomer 3	C17H20O9	367.1016	7.28	38.7	0.7	-4.98	98.51	PA	1.49E+05
6-hydroxyluteolin	C14H6O8	301.0005	7.41	37.5	0.0	4.90	93.41	F	7.67E+04
Viniferin isomer	C28H22O6	453.1368	7.46	36.0	0.0	5.33	86.27	S	3.21E+04
Flavonoid derivative 1	C15H12O5	271.0594	7.48	35.3	0.0	-6.60	83.97	F	7.49E+03
Apigenin 7-O-apiosyl-glucoside	C26H28O14	563.1388	7.50	50.8	63.3	-3.16	94.32	F	1.68E+06
Apigenin di-C-glucoside isomer 2	C27H30O17	625.1397	7.57	44.5	30.2	-2.12	94.77	F	3.39E+05
5-(3',4'-dihydroxyphenyl)-γ-valerolactone	C11H12O4	207.0642	7.61	35.9	0.0	-9.88	90.09	PA	5.28E+03
3,5,7-trihydroxyflavone	C15H10O5	269.0435	7.66	39.2	9.1	-7.47	95.30	F	1.05E+04
Apigenin 6-C-glucoside	C21H20O10	431.0971	7.66	47.0	42.8	-2.92	95.49	F	2.84E+05
Dihydroxy-methoxyisoflavone isomer 1	C16H12O5	283.0597	7.68	39.6	5.3	-5.12	98.67	F	9.84E+04
Hydroxy-O-desmethylangolensin isomer 1	C15H14O5	273.0754	7.69	37.6	0.0	-5.41	94.08	F	7.15E+04
Myricetin 3-O-glucoside	C21H20O13	479.0823	7.71	53.3	71.3	-1.69	97.17	F	1.99E+06
Naringenin 7-O-rutinoside	C27H32O14	579.1706	7.77	42.5	31.7	-2.26	83.32	F	5.28E+04
Flavonoid derivative 2	C27H30O17	625.1380	7.78	36.1	0.0	-4.91	86.23	F	1.93E+05
Eupatorin/cirsilineol	C18H16O7	343.0802	7.80	46.6	43.0	-6.31	96.92	F	5.12E+03
Naringin 4'-O-glucoside	C21H22O10	433.1118	7.80	45.2	38.5	-5.15	93.24	F	1.54E+05
Chrysoeriol 7-O-apiosyl-glucoside	C27H30O15	593.1501	7.82	50.5	56.6	-1.80	97.97	F	1.87E+06

Pelargonidin 3,5-O-diglucoside	C27H31ClO15	629.1261	7.82	38.0	0.0	-2.82	93.44	F	2.66E+05
Dihydroquercetin O-rhamnoside isomer 1	C21H22O11	449.1074	7.85	45.7	41.7	-3.31	90.62	F	1.70E+04
Quercetin 3-O-glucosyl-rhamnosyl-galactoside	C33H40O21	771.1984	7.87	45.5	35.9	-0.70	92.72	F	6.74E+06
Trihydroxyflavone isomer	C15H10O5	269.0438	7.93	43.9	36.8	-6.52	90.33	F	1.34E+04
Pseudobaptigenin	C16H10O5	281.0435	7.93	37.8	10.4	-7.16	86.40	F	2.62E+04
Dihydroxy-methoxyisoflavone isomer 2	C16H12O5	283.0593	7.93	46.5	42.9	-6.70	97.09	F	1.14E+05
Quercetin 3-O-rhamnosyl-rhamnosyl-glucoside	C33H40O20	755.2029	8.01	46.7	36.8	-1.49	98.63	F	9.23E+05
Rhamnetin	C16H12O7	315.0486	8.03	38.0	2.5	-7.63	95.88	F	5.57E+03
Procyanidin dimer B-type isomer 4	C27H30O14	577.1546	8.03	44.2	27.6	-2.97	96.92	F	1.07E+06
Genistin	C21H20O10	431.0968	8.07	44.8	29.9	-3.71	98.63	F	1.15E+06
**Ferulic acid**	**C10H10O4**	**193.0492**	**8.09**	**38.1**	**0.2**	**-7.62**	**98.84**	**PA**	**2.16E+04**
**Quercetin 3-O-rutinoside**	**C27H30O16**	**609.1454**	**8.09**	**48.5**	**44.7**	**-1.12**	**98.90**	**F**	**1.05E+07**
Quercetin 3-O-xylosyl-rutinoside	C32H38O20	741.1874	8.12	40.7	9.1	-1.31	95.93	F	1.09E+05
Eriodictyol	C15H12O6	287.0538	8.17	37.3	0.0	-7.98	95.31	F	5.08E+04
Dihydroquercetin O-rhamnoside isomer 2	C21H22O11	449.1082	8.17	42.2	17.8	-1.73	95.34	F	1.37E+04
Gardenin B	C19H18O7	357.0955	8.19	41.6	24.6	-6.96	91.33	F	3.60E+04
5,7-dihydroxyflavone	C15H10O4	253.0483	8.23	39.4	10.0	-9.34	97.08	F	9.41E+03
Naringenin	C15H12O5	271.0590	8.23	42.7	26.1	-8.04	96.49	F	6.93E+04
Isorhamnetin	C16H12O7	315.0489	8.23	45.0	38.0	-6.60	94.73	F	1.29E+04
(+)-catechin 5-gallate	C22H18O10	441.0808	8.23	44.8	31.9	-4.28	97.30	F	5.61E+06
**Ellagic acid**	**C14H6O8**	**300.9972**	**8.25**	**45.3**	**36.2**	**-5.95**	**97.05**	**PA**	**1.05E+06**
Quercetin 3-O-rhamnosyl-rhamnosyl-glucoside isomer 1	C33H40O20	755.2013	8.30	44.7	31.9	-3.57	95.58	F	1.64E+06
Luteolin 7-O-glucuronide	C21H18O12	461.0688	8.32	49.1	63.5	-8.21	90.94	F	1.89E+04
**Quercetin 3-O-glucoside**	**C21H20O12**	**463.0864**	**8.32**	**55.0**	**80.2**	**-3.88**	**99.27**	**F**	**5.00E+06**
Nobiletin	C21H22O8	401.1210	8.37	40.4	14.6	-7.95	96.25	F	2.66E+04
Tectoridin	C22H22O11	461.1060	8.43	42.6	29.1	-6.25	91.19	F	2.01E+04
4'-O-methylepigallocatechin	C16H16O7	319.0799	8.44	46.8	44.7	-7.49	97.88	F	9.97E+04
Rosmarinic acid	C18H16O8	359.0772	8.48	41.3	14.9	-0.06	91.52	PA	1.04E+04
Isorhamnetin 3-O-glucoside	C22H22O12	477.1009	8.50	40.0	12.6	-6.12	94.12	F	2.72E+04
Kaempferol 3-O-rutinoside	C27H30O15	593.1485	8.57	47.3	44.1	-4.52	97.50	F	4.71E+06
Violanone	C17H16O6	315.0848	8.59	40.4	20.6	-8.34	90.90	F	4.10E+04
Phloridzin	C21H24O10	435.1264	8.59	36.0	0.0	-7.42	88.32	F	4.35E+04
Quercetin 3-O-xyloside/quercetin 3-O-arabinoside	C20H18O11	433.0747	8.62	43.8	27.6	-6.66	99.20	F	7.43E+05
Quercetin 3-O-rhamnoside	C21H20O11	447.0903	8.62	48.6	52.0	-6.69	98.67	F	5.97E+05
Nepetin	C16H12O7	315.0484	8.66	37.3	0.0	-8.44	95.87	F	3.38E+04
Oleuropein	C25H32O13	539.1776	8.73	41.1	13.0	1.08	93.69	OP	4.90E+04
Kaempferol 3-O-glucoside	C21H20O11	447.0914	8.78	47.2	44.5	-4.11	96.43	F	2.68E+06
**p-coumaric acid**	**C9H8O3**	**163.0387**	**8.88**	**47.3**	**46.4**	**-8.06**	**98.92**	**PA**	**2.29E+05**
Isorhamnetin 3-O-galactoside	C22H22O12	477.1017	8.88	37.0	1.3	-4.58	89.19	F	1.29E+05
Diosmetin 7-O-rutinoside	C28H32O15	607.1645	8.88	45.5	50.5	-3.92	81.58	F	3.85E+04
Flavonoid derivative 3	C15H12O5	271.0587	8.91	45.6	40.4	-9.22	97.58	F	2.31E+04
Dihydroxy-methoxyisoflavone isomer 3	C16H12O5	283.0586	8.91	38.4	14.9	-9.06	87.07	F	9.41E+03
Dihydroquercetin	C15H12O7	303.0485	8.93	41.2	18.1	-8.45	97.45	F	1.31E+05
3,7-dimethylquercetin	C17H14O7	329.0650	8.94	47.3	51.4	-5.07	90.81	F	1.00E+04
Hydroxy-O-desmethylangolensin isomer 2	C15H14O5	273.0741	8.96	46.8	46.5	-9.86	98.12	F	8.37E+04
Naringin	C27H32O14	579.1698	9.01	38.7	5.2	-3.66	92.59	F	1.92E+04
Homoeriodictyol	C16H14O6	301.0696	9.25	37.4	0.0	-7.11	95.12	F	1.31E+04
7-hydroxymatairesinol	C20H22O7	373.1266	9.25	36.8	0.0	-7.12	91.95	L	3.16E+04
Piceatannol	C14H12O4	243.0642	9.28	36.6	7.4	-8.53	85.08	S	5.12E+03
**Myricetin**	**C15H10O8**	**317.0285**	**9.28**	**47.7**	**47.2**	**-5.77**	**98.04**	**F**	**4.11E+05**
Urolithin A	C13H8O4	227.0328	9.30	40.8	24.4	-9.43	89.81	OP	1.71E+04
Jaceosidin	C17H14O7	329.0638	9.32	36.3	0.0	-8.58	91.15	F	8.21E+03
Glycitin	C22H22O10	445.1138	9.41	38.6	7.4	-0.54	86.29	F	6.53E+03
Apigenin 7-O-glucoside	C21H24O9	419.1322	9.46	51.6	73.8	-6.12	90.96	F	2.45E+04
Flavonoid derivative 4	C15H12O5	271.0589	9.48	47.1	51.0	-8.58	93.98	F	1.72E+04
Trachelogenin	C21H24O7	387.1431	9.52	37.5	2.1	-4.67	90.80	L	5.73E+04
6-geranylnaringenin	C25H28O5	407.1829	9.57	35.8	3.5	-8.69	85.34	F	1.66E+04
Trihydroxy-dimethoxyflavone	C17H14O7	329.0646	9.59	37.9	0.0	-6.27	96.45	F	7.51E+03
3-O-methylviolanone	C18H18O6	329.1048	9.64	37.5	0.0	5.35	93.88	F	2.14E+04
Isohydroxymatairesinol	C20H22O7	373.1297	9.68	38.8	0.0	1.21	95.61	L	3.02E+04
Hesperetin	C16H14O6	301.0706	9.80	53.7	76.9	-3.91	96.38	F	1.41E+04
Theaflavin	C29H24O12	563.1177	9.87	43.4	23.3	-3.22	97.61	F	2.55E+05
Dihydrodaidzein/isoliquiritigenin	C15H12O4	255.0674	10.07	43.9	40.4	4.38	84.17	F	1.09E+04
Theaflavin 3-O-gallate	C36H28O16	715.1293	10.07	46.1	34.0	-1.65	98.40	F	7.21E+05
Luteolin 6-C-glucoside	C21H20O11	447.0910	10.14	37.7	2.3	-5.03	91.90	F	3.49E+04
Theaflavin 3,3'-O-digallate	C43H32O20	867.1413	10.14	43.4	22.3	-0.14	95.01	F	3.99E+05
**Quercetin**	**C15H10O7**	**301.0340**	**10.23**	**53.7**	**75.2**	**-4.60**	**98.70**	**F**	**2.45E+06**
Medioresinol	C21H24O7	387.1441	10.44	55.3	92.1	-2.24	87.14	L	1.16E+04
Paeoniflorin	C23H28O11	479.1569	10.44	42.3	26.8	2.21	87.19	PA	7.13E+03
Trihydroxyisoflavanone isomer	C15H12O5	271.0597	10.86	38.4	0.8	-5.54	97.48	F	7.18E+04

Matairesinol	C20H22O6	357.1325	10.89	36.3	0.0	-5.16	87.23	L	1.29E+04
**Apigenin**	**C15H10O5**	**269.0441**	**10.93**	**38.2**	**0.0**	**-5.25**	**96.85**	**F**	**4.25E+04**
Angolensin	C16H16O4	271.0993	10.98	37.1	0.0	6.24	92.59	F	4.67E+04
**Kaempferol**	**C15H10O6**	**285.0392**	**11.09**	**45.0**	**31.7**	**-4.56**	**98.57**	**F**	**7.42E+05**
Phloretin	C15H14O5	273.0757	11.10	48.5	55.4	-4.09	92.03	F	1.68E+04
Isotectorigenin	C16H12O6	299.0547	11.10	51.0	68.5	-4.64	91.97	F	2.65E+04
O-desmethylangolensin	C15H14O4	257.0808	11.45	35.8	0.0	-4.58	84.15	F	1.12E+04

Figure S2. Example of a 1500 kcal food plan.

Food Plan

Meals	Food	Quantity
**Breakfast**	Whole wheat bread<br>Crumble eggs<br>Black coffee without sugar or sweetener<br>Melon	2 slices (50g)<br>1 medium unit (50g)<br>½ cup (150ml)<br>1 small slice (90g)
**Morning snack**	Pear	1 average unit (130g)
**Lunch**	White rice<br>*Carioca* beans<br>Braised chicken fillet<br>Cooked carrots<br>Braised chayote<br>Tomato<br>Watercress<br>Roasted sweet potato	4 tablespoons (100g)<br>4 tablespoons (68g)<br>1 average unit (80g)<br>2 tablespoons (50g)<br>2 tablespoons (50g)<br>3 slices (45g)<br>3 branches (15g)<br>4 thin slices (140g)
**Afternoon snack**	Toasts<br>Tuna pâté<br>Orange	2 units (20g)<br>2 level tablespoons (30g)<br>1 average unit (180g)
**Dinner**	Omelet:<br>Egg<br>Spinach<br>Tomato<br>*Minas* Cheese	 2 medium units (100g)<br>2 tablespoons (40g)<br>3 slices (45g)<br>2 thin slices (68g)

Table S1 – CONSORT Checklist

CONSORT 2010 checklist of information to include when reporting a randomised trial*

Section/Topic	Item No	Checklist item	Reported on page No
**Title and abstract**			
	1a	Identification as a randomised trial in the title	1
	1b	Structured summary of trial design, methods, results, and conclusions (for specific guidance see CONSORT for abstracts)	1
**Introduction**			
Background and objectives	2a	Scientific background and explanation of rationale	3-4
	2b	Specific objectives or hypotheses	4
**Methods**			
Trial design	3a	Description of trial design (such as parallel, factorial) including allocation ratio	4-5
	3b	Important changes to methods after trial commencement (such as eligibility criteria), with reasons	5
Participants	4a	Eligibility criteria for participants	5
	4b	Settings and locations where the data were collected	4,9
Interventions	5	The interventions for each group with sufficient details to allow replication, including how and when they were actually administered	6-8
Outcomes	6a	Completely defined pre-specified primary and secondary outcome measures, including how and when they were assessed	4; 8-11
	6b	Any changes to trial outcomes after the trial commenced, with reasons	-
Sample size	7a	How sample size was determined	6
	7b	When applicable, explanation of any interim analyses and stopping guidelines	-
Randomisation:			
Sequence generation	8a	Method used to generate the random allocation sequence	6
	8b	Type of randomisation; details of any restriction (such as blocking and block size)	6
Allocation concealment mechanism	9	Mechanism used to implement the random allocation sequence (such as sequentially numbered containers), describing any steps taken to conceal the sequence until interventions were assigned	6

Implementation	10	Who generated the random allocation sequence, who enrolled participants, and who assigned participants to interventions	6
Blinding	11a	If done, who was blinded after assignment to interventions (for example, participants, care providers, those assessing outcomes) and how	-
	11b	If relevant, description of the similarity of interventions	-
Statistical methods	12a	Statistical methods used to compare groups for primary and secondary outcomes	11-12
	12b	Methods for additional analyses, such as subgroup analyses and adjusted analyses	11-12
**Results**			
Participant flow (a diagram is strongly recommended)	13a	For each group, the numbers of participants who were randomly assigned, received intended treatment, and were analysed for the primary outcome	13
	13b	For each group, losses and exclusions after randomisation, together with reasons	13
Recruitment	14a	Dates defining the periods of recruitment and follow-up	13
	14b	Why the trial ended or was stopped	-
Baseline data	15	A table showing baseline demographic and clinical characteristics for each group	Table 2,p.30
Numbers analysed	16	For each group, number of participants (denominator) included in each analysis and whether the analysis was by original assigned groups	13
Outcomes and estimation	17a	For each primary and secondary outcome, results for each group, and the estimated effect size and its precision (such as 95% confidence interval)	13-15
	17b	For binary outcomes, presentation of both absolute and relative effect sizes is recommended	-
Ancillary analyses	18	Results of any other analyses performed, including subgroup analyses and adjusted analyses, distinguishing pre-specified from exploratory	-
Harms	19	All important harms or unintended effects in each group (for specific guidance see CONSORT for harms)	-
**Discussion**			
Limitations	20	Trial limitations, addressing sources of potential bias, imprecision, and, if relevant, multiplicity of analyses	19
Generalisability	21	Generalisability (external validity, applicability) of the trial findings	15-19
Interpretation	22	Interpretation consistent with results, balancing benefits and harms, and considering other relevant evidence	15-19
**Other information**			
Registration	23	Registration number and name of trial registry	6
Protocol	24	Where the full trial protocol can be accessed, if available	-
Funding	25	Sources of funding and other support (such as supply of drugs), role of funders	21

Citation: Schulz KF, Altman DG, Moher D, for the CONSORT Group. CONSORT 2010 Statement: updated guidelines for reporting parallel group randomised trials. BMC Medicine. 2010;8:18.
© 2010 Schulz et al. This is an Open Access article distributed under the terms of the Creative Commons Attribution License (http://creativecommons.org/licenses/by/2.0), which permits unrestricted use, distribution, and reproduction in any medium, provided the original work is properly cited.
